# Exploring the efficacy of Transcutaneous Auricular Vagus nerve stimulation (taVNS) in modulating local and systemic inflammation in experimental models of colitis

**DOI:** 10.1186/s42234-024-00162-5

**Published:** 2024-12-09

**Authors:** Fatemeh Hesampour, Diane M Tshikudi, Charles N Bernstein, Jean-Eric Ghia

**Affiliations:** 1 Immunology, Rady Faculty of Health Sciences, Max Rady College of Medicine, University of Manitoba, Winnipeg, Canada; 2 Internal Medicine Section of Gastroenterology, Rady Faculty of Health Sciences, Max Rady College of Medicine, University of Manitoba, Winnipeg, Canada; 3 Inflammatory Bowel Disease Clinical & Research Centre, Rady Faculty of Health Sciences, Max Rady College of Medicine, University of Manitoba, Winnipeg, Canada; 4 Children’s Hospital Research Institute of Manitoba, Winnipeg, Canada; 5 Department of Immunology, Internal Medicine Section of Gastroenterology, Apotex Centre 431, 750 McDermot Avenue, Winnipeg, MB R3E 0T5 Canada

**Keywords:** Non-invasive vagus nerve stimulation, Vagus nerve stimulation, Inflammatory bowel disease, Colitis, Bioelectronic medicine, Local anti-inflammatory effects, Systemic anti-inflammatory effects, Transcutaneous auricular vagus nerve stimulation

## Abstract

**Background:**

Current inflammatory bowel disease (IBD) treatments often fail to achieve lasting remission and have adverse effects. Vagus nerve stimulation (VNS) offers a promising therapy due to its anti-inflammatory effects. Its invasive nature, however, has led to the development of non-invasive methods like transcutaneous auricular VNS (taVNS). This study assesses taVNS’s impact on acute colitis progression, inflammatory, anti-inflammatory, and apoptosis-related markers.

**Methods:**

Male C57BL/6 mice (11–12 weeks) were used for dextran sulfate sodium (DSS)- and dinitrobenzene sulfonic acid (DNBS)-induced colitis studies. The administration of taVNS or no stimulation (anesthesia without stimulation) for 10 min per mouse began one day before colitis induction and continued daily until sacrifice. Ulcerative colitis (UC)-like colitis was induced by administering 5% DSS in drinking water for 5 days, after which the mice were sacrificed. Crohn’s disease (CD)-like colitis was induced through a single intrarectal injection of DNBS/ethanol, with the mice sacrificed after 3 days. Disease activity index (DAI), macroscopic evaluations, and histological damage were assessed. Colon, spleen, and blood samples were analyzed via qRT-PCR and ELISA. One-way or two-way ANOVA with Bonferroni and Šídák tests were applied.

**Results:**

taVNS improved DAI, macroscopic, and histological scores in DSS colitis mice, but only partially mitigated weight loss and DAI in DNBS colitis mice. In DSS colitis, taVNS locally decreased colonic inflammation by downregulating pro-inflammatory markers (IL-1β, TNF-α, Mip1β, MMP 9, MMP 2, and Nos2) at the mRNA level and upregulating anti-inflammatory TGF-β in non-colitic conditions at both mRNA and protein levels and IL-10 mRNA levels in both non-colitic and colitic conditions. Systemically, taVNS decreased splenic TNF-α in non-colitic mice and increased serum levels of TGF-β in colitic mice and splenic levels in non-colitic and colitic mice. Effects were absent in DNBS-induced colitis. Additionally, taVNS decreased pro-apoptotic markers (Bax, Bak1, and caspase 8) in non-colitic and colitic conditions and increased the pro-survival molecule Bad in non-colitic mice.

**Conclusions:**

This study demonstrates that taVNS has model-dependent local and systemic effects, reducing inflammation and apoptosis in UC-like colitis while offering protective benefits in non-colitic conditions. These findings encourage further research into underlying mechanisms and developing adjunct therapies for UC.

## Background

Inflammatory bowel disease (IBD), which comprises ulcerative colitis (UC) and Crohn’s disease (CD), is characterized by chronic inflammation of the digestive tract without an established cause (Hanauer 2024). This condition is associated with ulcerative lesions, excessive death of intestinal epithelial cells, an imbalance in pro-apoptotic and pro-survival molecules, and heightened tissue damage due to inflammatory mediators secreted by immune cells (Singh et al. 2016). Paralleling local dysregulations, a decrease in the parasympathetic nervous system has been described, particularly the vagus nerve activity, with studies finding alterations in the size and number of autonomic nerves, leading to impaired parasympathetic functioning in IBD patients (Gondim et al. 2005, Ananthakrishnan et al. 2010, Pellissier et al. 2014). Anti-tumor necrosis factor (TNF) therapy is one of the IBD treatments (Baert et al. 1999). It limits T-cell proliferation, and the detrimental consequences of cytokines produced by innate and adaptive immune cells within the lamina propria, indirectly restoring gut barrier function and increasing mucosal secretions (Ungar et al. 2016). However, studies have shown that 10–30% of patients experience primary non-response, and 23–46% of individuals lose responsiveness over time. The annual risk of response loss to anti-TNF therapy has been documented as 13% for infliximab and nearly 20% for adalimumab (Roda et al. 2016). Discovery of a low vagal tone linked to a high plasma level of TNF-α has propelled the hypothesis of a specific relationship between TNF-α levels and vagal tone in IBD patients (Pellissier et al. 2014). Furthermore, over the last 20 years, the vagus nerve has emerged as a focus of bioelectronic medicine due to its precise anatomical link between the brain and gut, and its integral function within the neuroendocrine-immune axis (Bonaz et al. 2021). Acetylcholine (ACh) and central VNS using galantamine have been shown to possess potential anti-inflammatory effects on the function of intestinal immune cells and the production of inflammatory cytokines, such as TNF-α and interleukin (IL)−1β, through the splenic nerve and spleen via the α7 nicotinic ACh receptor (α7nAChR) (Zheng et al. 2024, Ji et al. 2014, Wang et al. 2003). Traditionally, VNS has been administered through invasive procedures requiring the surgical implantation of electrodes at the cervical region, a method approved by the U.S. Food and Drug Administration (FDA) for managing epilepsy and depression (Rush et al. 2000, Groves and Brown 2005). Invasive VNS has shown promise in reducing inflammation and improving clinical outcomes in patients with CD and UC. Studies report significant reductions in disease activity, fecal calprotectin, and inflammatory markers, along with improvements in pain (Bonaz et al. 2016, Sinniger et al. 2020, D’Haens et al. 2023). Additionally, invasive VNS has demonstrated therapeutic potential in animal models of IBD (Meroni et al. 2018, Sun et al. 2013, Payne et al. 2019, Meroni et al. 2021, Meregnani et al. 2011). Nevertheless, cervical-level VNS poses potential risks due to the proximity of the vagus nerve to critical vascular structures, such as the external carotid artery and the jugular vein, which may result in undesirable side effects (Bonaz et al. 2016). Given that, a non-invasive and drug-free procedure called non-invasive transcutaneous auricular vagus nerve stimulation (taVNS) has been developed and has gained attention as a complementary therapy for inflammatory and TNF-mediated conditions (Badran et al. 2018). A recent trial on taVNS in young IBD patients has shown a reduction in fecal calprotectin in a proportion of participants, with clinical remission in CD and UC patients. Improvements in quality of life were also observed, with no reported safety concerns (Sahn et al. 2023). It is thought that taVNS stimulates the nucleus tractus solitarii (NTS) in the brainstem’s medulla (Badran et al. 2018, Serafini et al. 2022). This activation then triggers the central circuitry of the dorsal motor nucleus of the vagus nerve (DMV), the activation of efferent vagal nerve fibers, known to activate the cholinergic anti-inflammatory pathway (CAP or CAIP), and eventually the enteric nervous system (ENS) to restore gut homeostasis and function (Badran et al. 2018, Serafini et al. 2022, Rosas-Ballina et al. 2008). Nevertheless, this effect of the vagus nerve is indirect, as it is mediated by the interaction of the vagus nerve with enteric neurons located in the small intestine and proximal part of the colon muscularis (Mikami et al. 2022). These neurons, which express choline acetyltransferase (ChAT) and ACh, have nerve terminals near certain immune cells, such as resident muscularis macrophages (MMØs) that express α7nAChR. This arrangement is referred to as the intestinal CAP (Cailotto et al. 2014). Furthermore, a second route via the spleen exists and is involved in the anti-inflammatory effects of the vagus nerve, with the spleen producing a considerable level of TNF-α and ACh in the body (Tracey 2007). This process, known as the splenic CAP, which is non-neuronal, is regulated by a vagal-splenic pathway that links the spleen and the vagal efferent endings through the celiac sympathetic ganglion (Mikami et al. 2022). The vagus nerve is involved in the neuroimmune axis by engaging afferent and efferent fibers (Bonaz et al. 2017). Besides activating the CAP, the vagus nerve, through its afferent fibers, also triggers the hypothalamic-pituitary-adrenal (HPA) axis, prompting the adrenal glands to release glucocorticoids (Bonaz et al. 2017). Martelli et al. challenged the original CAP model, arguing that the vagus nerves did not regulate inflammation in their endotoxaemic animal model (Martelli et al. 2016). Their research indicated that the efferent motor pathway, termed the ‘splanchnic anti-inflammatory pathway,’ is exclusively sympathetic, transmitted via the greater splanchnic nerves, which activate postganglionic sympathetic neurons to modulate the inflammatory response to immune challenges (Martelli et al. 2016). They also proposed that the vagus nerves are likely not involved in this reflex, as vagotomy, disrupting both afferent and efferent fibers, did not appear to affect the inflammation induced by endotoxemia (Martelli et al. 2016).

Given that, we hypothesized that taVNS, when administered to UC- and CD-like preclinical mouse models, could decrease the development of acute colitis in mice by improving the disease activity index, decreasing pro-inflammatory markers, increasing anti-inflammatory cytokines, and harnessing apoptotic markers.

## Methods

### Animal

Male C57BL/6 mice, aged 11–12 weeks and weighing 22–28 g, were obtained from Charles River (Sherbrook, Canada) or Central Animal Care Services (CACS) (Bannatyne Campus, University of Manitoba). These animals were housed in pathogen-free facilities at the University of Manitoba’s animal care center. All procedures complied with the approved protocol (Protocol Number: 20–064) by the University of Manitoba Animal Ethics Committee.

### Induction of dextran sulfate sodium (DSS)-induced acute colitis and taVNS stimulation

Thirty-two male C57BL/6 mice were divided into four groups, DSS-taVNS, DSS-CTRL, CTRL-taVNS, and CTRL-CTRL, with eight mice per group based on DSS treatment and taVNS or no stimulation (anesthesia without any stimulation). After anesthetizing each mouse, the auricular nerve stimulation device (Vagustim Animal Research Device (ARD), Vagustim, Istanbul, Turkey) with preset stimulation parameters (voltage, 10 V, Intensity, 1 mA; Pulse Width, 500 µs; on/off Duration, 30 s; stimulation duration, 10 min; frequency, 20 Hz) was connected to two electrodes placed in the cymba concha of two ears in CTRL-taVNS and DSS-taVNS groups. taVNS and no stimulation were initiated one day before the induction of acute colitis and continued daily for all mice until sacrifice, after six days of taVNS or no stimulation. After 24 h, colitis was induced by adding 5% DSS (wt/vol) (molecular weight, 40 kDa; Thermo Fisher Scientific, Canada) to drinking water of the DSS-CTRL and DSS-taVNS for five days, and the CTRL-CTRL and CTRL-taVNS mice continued to receive regular water. The mean DSS consumption per cage was recorded each day. The composite disease activity index (DAI) (weight loss percentage, feces bleeding, and stool consistency loss) was recorded during this period. Scoring was based on the following criteria: weight loss (0 for no loss, 1 for 1–5%, 2 for 5–10%, 3 for 10–20%, and 4 for > 20%); stool consistency (0 for normal and 2 for loose stool, and 4 for diarrhea); and bleeding (0 for no blood, 1 for hemoccult positive, 2 for positive fecal occult blood test with observed pellet bleeding in stool, and 4 for visible rectal bleeding with blood present around the anal region) (Melgar et al. 2005). Colon, spleen, and blood samples were obtained on the day of sacrifice. To assess the effects of taVNS on acute colitis induction, colon length and macroscopic scores were analyzed. Tissue samples from the distal colon were preserved in formalin, embedded in paraffin, sectioned into 4 μm slices, and stained with hematoxylin and eosin for histological analysis (Thermo Fisher Scientific, Canada). The score for histological damage was calculated based on the following factors: goblet cell depletion (0–1 scale, 0 representing absence and 1 representing the presence of goblet cell depletion), crypt abscess (0–1 scale, 0 representing absence and 1 representing crypt abscess), muscle thickening (0–3 scale, 0 representing the base of the crypt on the muscularis mucosae and 3 representing muscle thickening), crypt architecture (0–3 scale, 0 representing normal crypt architecture and 3 representing significant crypt architectural distortion with complete loss of some crypt structures), and extent of inflammatory cell infiltration (0–3 scale, 0 representing normal and 3 representing dense inflammatory infiltrate) (Melgar et al. 2005).

### Induction of Dinitrobenzene sulfonic acid (DNBS)-induced colitis

Thirty-two, 11–12-week-old male C57BL/6 mice were divided into four groups, DNBS-taVNS, DNBS-CTRL, ETOH-taVNS, or ETOH-CTRL, based on whether they received DNBS/ethanol treatment and taVNS/no stimulation. One day before the induction of DNBS-induced colitis, taVNS mice received 10 min/daily stimulation with no stimulation (anesthesia without any stimulation) for CTRL mice, which lasted until the end of the experiment. After 24 h, mice received a single dose (100 µL) of intrarectal 30% ethanol or DNBS (4 mg/mouse) dissolved in 30% ethanol. Intrarectal injections were carried out using PE-90 tubing, supplied by ClayAdam (Parsippany, NJ, United States), which was inserted 3.5 cm into the colon and connected to a tuberculin syringe manufactured by BD (Mississauga, ON, Canada). On day 5, all mice were euthanized, and colon tissue, spleen, and blood samples were collected.

### Colonic, Splenic, and serum protein assay

Colonic and splenic samples were homogenized mechanically using an ultrasonic processor (PRO Scientific Inc., USA) in 500 µL of PBS containing protease inhibitors (Sigma-Aldrich, Canada), centrifuged for 20 min, and the supernatants were frozen at −20 °C until assay. The Bradford protein assay (Sigma-Aldrich, Canada) quantified the protein concentration in tissue homogenates. Colonic, splenic, and serum levels of different inflammatory and anti-inflammatory cytokines were measured by enzyme-linked immunosorbent assay (ELISA). Commercial ELISA kits for IL-10, transforming growth factor (TGF)-β1, TNF-α (R&D Systems, Inc., MN, USA), IL-1β (Biolegend, USA), and mouse corticosterone (Enzo Life Sciences, Inc., USA) were used.

### Quantitative real-time reverse-transcription polymerase chain reaction (qRT-PCR)

The distal colon tissue was minced into 1 ml TRIzol™ and homogenized using an ultrasonic processor (PRO Scientific Inc., USA). Total RNA was extracted and purified using an RNA purification kit (Life Technologies, NY, USA), followed by reverse transcription using the SuperScript VILO cDNA Synthesis Master Mix (Invitrogen, NY, USA). Real-time quantitative PCR was employed for gene expression quantification using Power SYBR Green Master Mix (Life Technologies, Burlington, ON, Canada) on a Roche Light Cycler 96 Real-Time System. The differences between the target genes and mouse TATA-box binding protein (TBP) were calculated and normalized based on Roche Light Cycler calculation method (Pfaffl 2001). The inflammatory and anti-inflammatory markers measured in association with the development of colitis in mice are listed in Table1, along with their corresponding primer sequences, which were used to measure their expression levels.

**Table 1:** Primers sequences

Gene Name	Forward (5ʹ−3ʹ)	Reverse (5ʹ−3ʹ)
Nos2	GTTCTCAGCCCAACAATACAAGA	GTGGACGGGTCGATGTCAC
Mip1β	TTCCTGCTGTTTCTCTTACACCT	CTGTCTGCCTCTTTTGGTCAG
Il1b	GCAACTGTTCCTGAACTCAACT	ATCTTTTGGGGTCCGTCAACT
Tnf	CCCTCACACTCAGATCATCTTCT	GCTACGACGTGGGCTACAG
Mmp2	GATAACCTGGATGCCGTCGTG	GGTGTGCAGCGATGAAGATGATA
Mmp9	GCCCTGGAACTCACACGACA	TTGGAAACTCACACGCCAGAAG
Il10	GCTCTTACTGACTGGCATGAG	CGCAGCTCTAGGAGCATGTG
Tgfb1	TGACGTCACTGGAGTTGTACGG	GGTTCATGTCATGGATGGTGC
Bad	AAGTCCGATCCCGGAATCC	GCTCACTCGGCTCAAACTCT
Bak1	CAACCCCGAGATGGACAACTT	CGTAGCGCCGGTTAATATCAT
Bax	TGAAGACAGGGGCCTTTTTG	AATTCGCCGGAGACACTCG
Casp8	CAACTTCCTAGACTGCAACCG	TCCAACTCGCTCACTTCTTCT
TBP	CGTGAATCTTGGCTGTAAACT	GTCCGTGGCTCTCTTATTCT

*Nos* Nitric oxide synthase, *Mip* Macrophage inflammatory protein, *IL* Interleukin, *TNF* Tumor necrosis factor, *MMP*: Matrix metalloproteinases, *TGF* Transforming growth factor, *Bad* BCL2 associated agonist of cell death, *Bak1* BCL2 antagonist/killer 1, *Bax*: Bcl-2-associated protein x, *Casp8* Caspase 8, *TBP* TATA-box binding protein

#### Data analysis

The differences between groups were assessed using either one-way or two-way ANOVA alongside multiple parametric comparisons (Bonferroni and Šídák), facilitated by GraphPad Prism software (version 9; GraphPad Software, Inc., La Jolla, CA, USA). Percentage differences between groups in mRNA and protein levels of inflammatory and anti-inflammatory markers were calculated from the data prior to normalization. The significance level was set at *P*-values (two-tailed) below 0.05. The data is presented as the mean ± SD. For animal experiments, a sample size of *n* = 8 was used to achieve 85% power with a 95% confidence interval based on a test value of 50%, accounting for maximum variability.

## Results

### Disease activity index, macroscopic scores, and histological scores in DSS-induced acute colitis

**Fig. 1:**
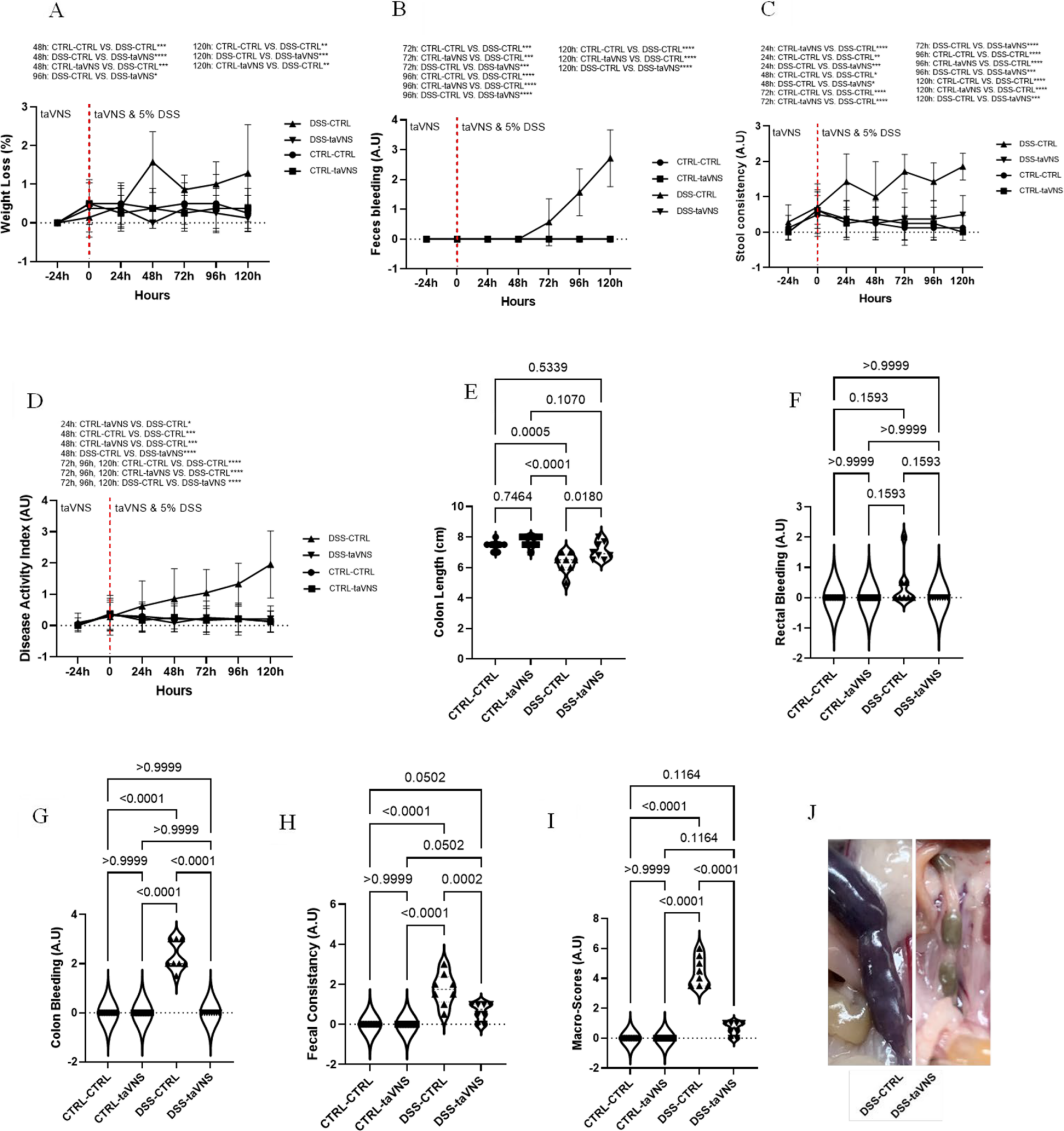
Non-invasive transcutaneous auricular vagus nerve stimulation (taVNS) improves the colitis-related disease activity index (DAI) and macroscopic score. taVNS (with stimulation parameters of 10 volts, a 500 µs pulse width, 30 s on/off duration, 10 min stimulation duration, and a frequency of 20 Hz) and no stimulation (anesthesia without any stimulation) were performed for the taVNS and CTRL (no stimulation) groups, respectively, beginning one day before the induction of acute colitis and continued daily until the mice were sacrificed. After 24 h, acute colitis was induced in the colitis (dextran sulfate sodium (DSS)) groups by adding 5% DSS to their drinking water for five days, while control groups continued to receive regular water. Percentage of weight loss (**A**), feces bleeding (**B**), stool consistency loss (**C**), disease activity index (**D**), colon length (**E**), rectal bleeding (**F**), colon bleeding (**G**), fecal consistency (**H**), and macroscopic score (**I**-**J**). One-way and Two-way ANOVA and multiple parametric comparison tests (Bonferroni and Šídák) were used to calculate *P* values. The significance level was set at *p* < 0.05. *n* = 7–8 mice/group. Each value is presented as the mean ± SD. This experiment was repeated at least four times

As shown in Fig. 1A, DSS-taVNS and non-colitic mice experienced significantly less weight loss than DSS-CTRL mice from 48 h until the end of the experiment. There was no evidence of blood in the feces of DSS-taVNS, CTRL-CTRL, or CTRL-taVNS mice. Still, a significant presence of blood was identified in the feces of DSS-CTRL mice, starting from the time point of 72 h by the end of the experiment (Fig. 1B). Starting at 24 h, the DSS-taVNS and non-colitic groups showed a considerably less stool consistency loss compared to the DSS-CTRL mice (Fig. 1C). By the 48-hour time point and continuing until the end of the experiment, the DSS-taVNS mice had a considerably lower DAI than the DSS-CTRL mice (Fig. 1D). DSS colitis induced a significant decrease of colon length, and this effect was abolished in DSS-taVNS mice (Fig. 1E). In addition, three macroscopic parameters, including rectal bleeding (Fig. 1F), colon bleeding (Fig. 1G), and fecal consistency (Fig. 1H), were assessed upon sacrifice. Although no modification was observed in rectal bleeding, DSS-taVNS mice showed significantly less colon bleeding and fecal consistency loss than DSS-CTRL mice. A substantial difference was also observed between DSS-taVNS and DSS-CTRL when the composition of the three parameters was used to calculate and compare the total macroscopic scores (Fig. 1I and Image 1 J).

**Fig. 2:**
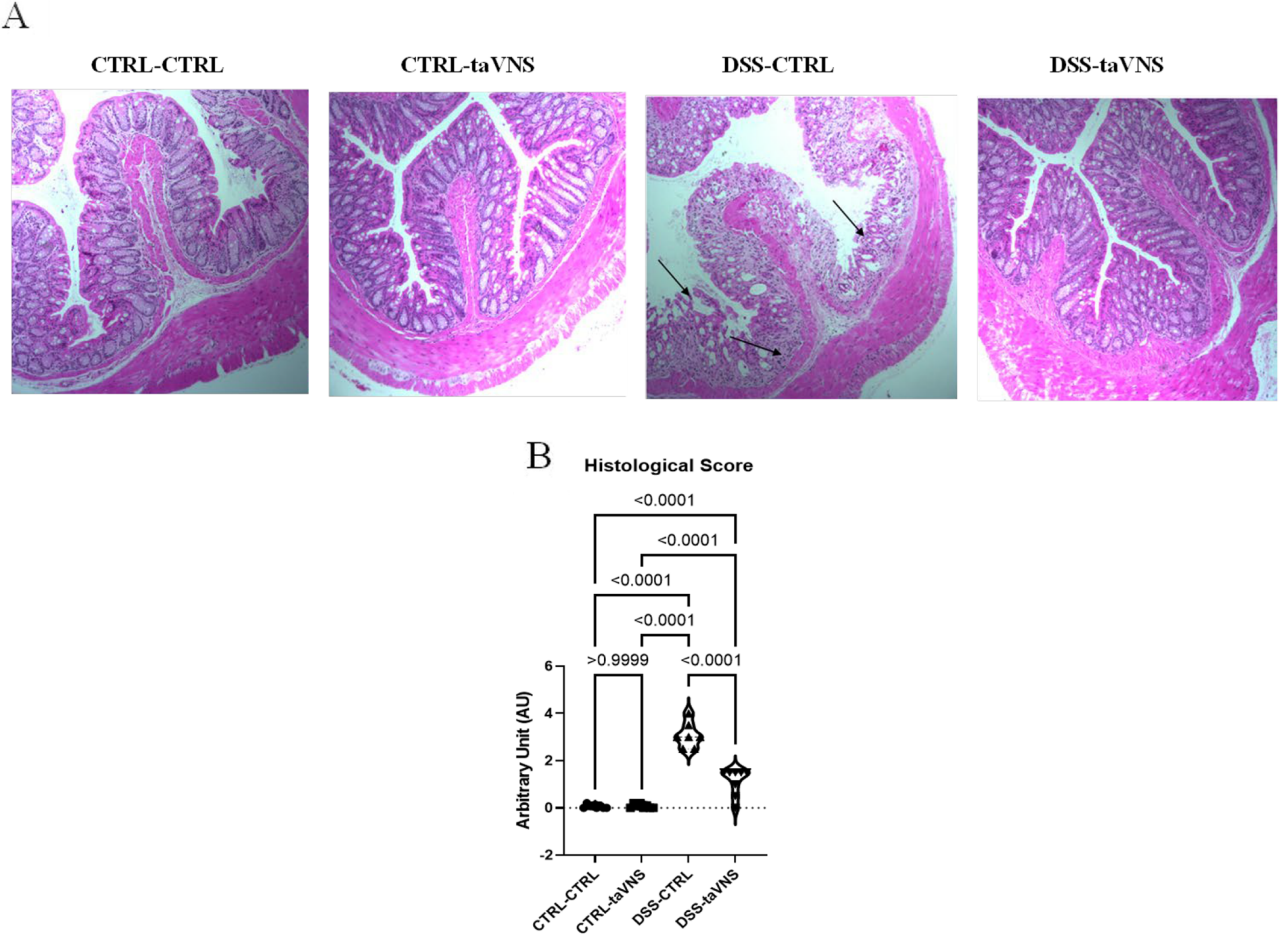
Non-invasive transcutaneous auricular vagus nerve stimulation (taVNS) improves the colitis-related histological score. taVNS (with stimulation parameters of 10 volts, a 500 µs pulse width, 30 s on/off duration, 10 min stimulation duration, and a frequency of 20 Hz) and no stimulation (anesthesia without any stimulation) were performed for the taVNS and CTRL (no stimulation) groups, respectively, beginning one day before the induction of acute colitis and continued daily until the mice were sacrificed. After 24 h, acute colitis was induced in the colitis (dextran sulfate sodium (DSS)) groups by adding 5% DSS to their drinking water for five days, while control groups continued to receive regular water. Histological appearance of hematoxylin and eosin-stained colonic tissue (40 × magnification) (**A**) and histological score (**B**). One-way ANOVA and multiple parametric comparison tests (Bonferroni and Šídák) were used to calculate *P* values. The significance level was set at *p* < 0.05. *n* = 8 mice/group. Each value is presented as the mean ± SD. This experiment was repeated at least four times

DSS-CTRL mice showed more severe architectural damage and more significant infiltration of immune cells into the inflamed gut (Image 2 A). Evaluations showed that non-colitic and DSS-taVNS mice displayed very few indications of mucosal damage, crypt loss, or inflammation, with assessment of the histological score revealing significantly lower scores in DSS-taVNS mice (Fig. 2B).

In summary, taVNS improved disease activity index, macroscopic scores, and histological scores in DSS-induced acute colitis.

### Local anti-inflammatory effects of taVNS in DSS-induced acute colitis

**Fig. 3:**
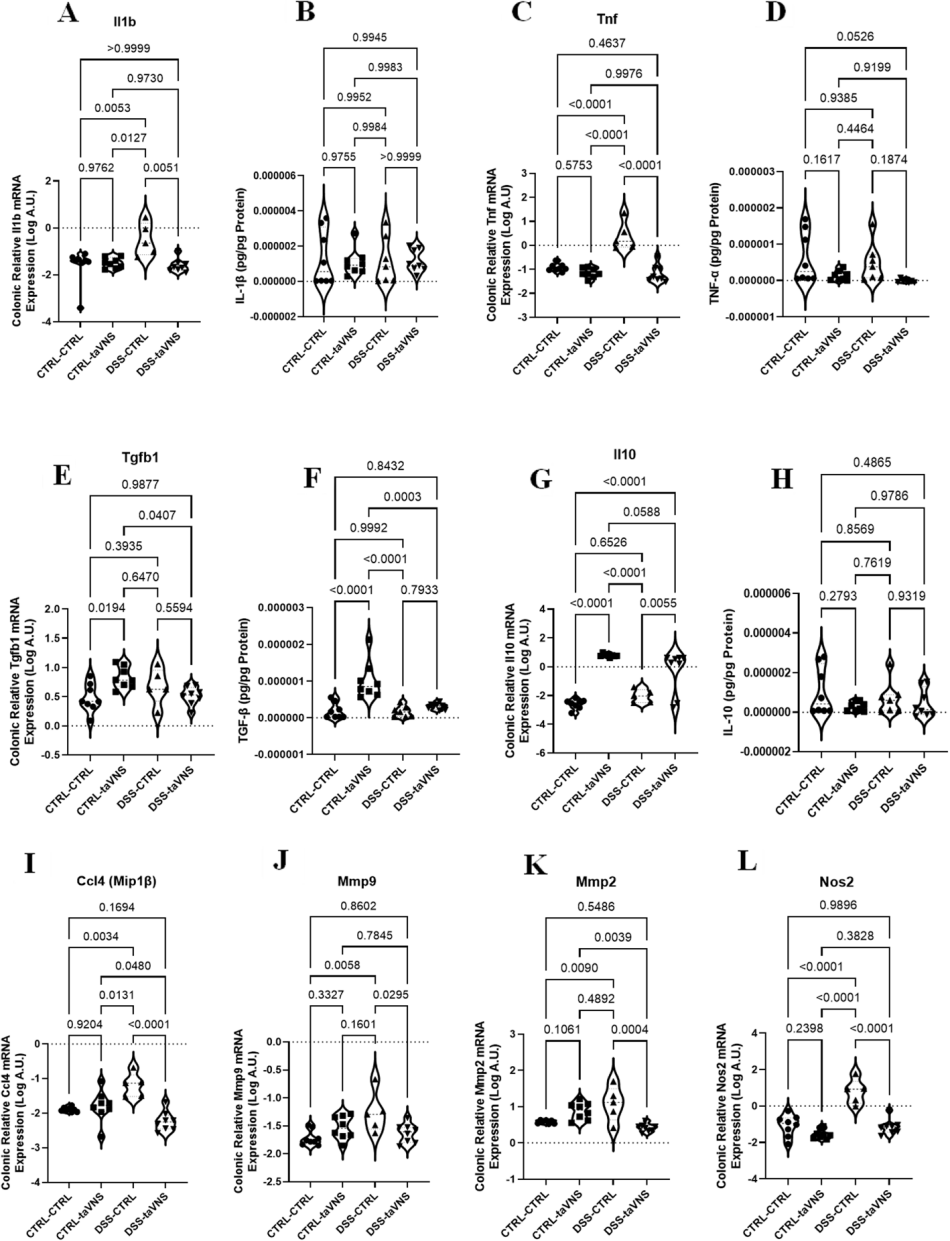
Local effects: Non-invasive transcutaneous auricular vagus nerve stimulation (taVNS) decreases colonic expression levels of pro-inflammatory markers in colitic mice and increases anti-inflammatory markers in non-colitic and colitic mice. taVNS (with stimulation parameters of 10 volts, a 500 µs pulse width, 30 s on/off duration, 10 min stimulation duration, and a frequency of 20 Hz) and no stimulation (anesthesia without any stimulation) were performed for the taVNS and CTRL (no stimulation) groups, respectively, beginning one day before the induction of acute colitis and continued daily until the mice were sacrificed. After 24 h, acute colitis was induced in the colitis (dextran sulfate sodium (DSS)) groups by adding 5% DSS to their drinking water for five days, while control groups continued to receive regular water. Colonic mRNA (**A**) and protein (**B**) levels of interleukin (IL)−1β, mRNA (**C**) and protein (**D**) levels of tumor necrosis factor (TNF)-α, mRNA (**E**) and protein (**F**) levels of transforming growth factor (TGF)-β, and mRNA (**G**) and protein (**H**) levels of IL-10, mRNA levels of macrophage inflammatory protein 1β (Mip1β) or chemokine (C-C motif) ligand 4 (CCL4) (**I**), matrix metalloproteinases 9 (MMP9) (**J**), MMP2 (**K**), and nitric oxide synthase 2 (Nos2) (**L**). One-way ANOVA and multiple parametric comparison tests (Bonferroni and Šídák) were used to calculate *P* values. The significance level was set at *p* < 0.05. *n* = 6–8 mice/group. Each value is presented as the mean ± SD. This experiment was repeated at least four times

The mRNA and protein expression of cytokines, chemokines, MMPs, and neutrophil/macrophage-associated markers were analyzed in colon tissue from all four mouse groups to evaluate the potential local effects of taVNS on acute colitis. taVNS significantly decreased the relative mRNA expression of IL-1β (Fig. 3A, 3.85%) and TNF-α (Fig. 3C, 1.66%) in colitic mice without modifying their protein levels (Fig. 3B and D), with no changes in non-colitic mice at either the mRNA or the protein level. Although no significant changes in colitic mice were seen, taVNS significantly increased the expression of the anti-inflammatory cytokine TGF-β1 at both the mRNA and protein levels in non-colitic conditions (Figs. 3E and F and 110.54% and 433.08%, respectively). taVNS significantly increased the mRNA expression of IL-10 in colitic and non-colitic mice (Figs. 3G and 17237.48% and 202025%, respectively); however, its protein levels were not modified (Fig. 3H).taVNS significantly decreased the colonic mRNA levels of Mip1β (CCL4) (Figs. 3I and 8.65%), MMP9 (Figs. 3J and 32.92%), MMP2 (Figs. 3K and 14.45%), and Nos2 (Fig. 3L, 0.78%) in colitic mice, with no modifications in non-colitic conditions.

In summary, taVNS demonstrated local anti-inflammatory effects by downregulating colonic pro-inflammatory markers under colitic conditions and upregulating anti-inflammatory markers in colitic and non-colitic mice.

### Systemic and splenic anti-inflammatory effects of taVNS in DSS-induced acute colitis

**Fig. 4:**
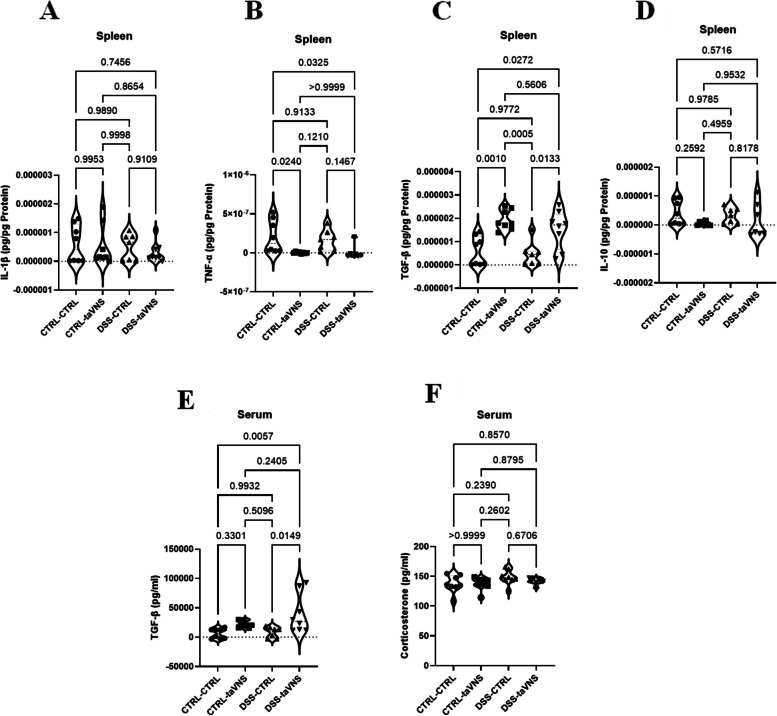
Systemic effects: Non-invasive transcutaneous auricular vagus nerve stimulation (taVNS) decreases splenic levels of tumor necrosis factor (TNF)-α in non-colitic mice and increases splenic and serum levels of transforming growth factor (TGF)-β in both non-colitic and colitic mice. taVNS (with stimulation parameters of 10 volts, a 500 µs pulse width, 30 s on/off duration, 10 min stimulation duration, and a frequency of 20 Hz) and no stimulation (anesthesia without any stimulation) were performed for the taVNS and CTRL (no stimulation) groups, respectively, beginning one day before the induction of acute colitis and continued daily until the mice were sacrificed. After 24 h, acute colitis was induced in the colitis (dextran sulfate sodium (DSS)) groups by adding 5% DSS to their drinking water for five days, while control groups continued to receive regular water. Splenic protein levels of interleukin (IL)−1β (**A**), TNF-α (**B**), TGF-β (C), IL-10 (**D**), and serum levels of TGF-β (**E**) and corticosterone (**F**). One-way ANOVA and multiple parametric comparison tests (Bonferroni and Šídák) were used to calculate P values. The significance level was set at *p* < 0.05. *n* = 7–8 mice/group. Each value is presented as the mean ± SD. This experiment was repeated at least four times

Splenic protein and serum levels of the pro-inflammatory and anti-inflammatory cytokines IL-1β, TNF-α, TGF-β, and IL-10 were assessed to evaluate the probable systemic effect of taVNS in DSS-induced acute colitis. taVNS did not change splenic IL-1β levels in mice (Fig. 4A). However, while there were no modifications in colitic mice, it significantly decreased splenic TNF-α levels in non-colitic mice (Fig. 4B, 0.86%). Although taVNS increased splenic levels of anti-inflammatory TGF-β in colitic and non-colitic mice (Figs. 4C and 226.27% and 223.78%, respectively), splenic IL-10 levels showed no significant changes (Fig. 4D).

taVNS significantly increased serum TGF-β levels in colitic conditions when compared to non-stimulated colitic conditions, without modifying non-colitic mice (Figs. 4E and 359.49%). In addition, to test the possible involvement of the HPA axis in the systemic anti-inflammatory effects of taVNS, the serum concentration of corticosterone, a hormone released by the adrenal glands following activation of the HPA axis (Bonaz 2017), was measured, as indicated in Fig.4F, and no significant differences were observed in mice.

In conclusion, taVNS demonstrated systemic anti-inflammatory effects by decreasing splenic TNF-α levels in non-colitic mice, while simultaneously increasing splenic and serum TGF-β levels in both non-colitic and colitic mice.

### The colonic expression levels of pro- and anti-apoptotic markers in DSS-induced acute colitis

**Fig. 5:**
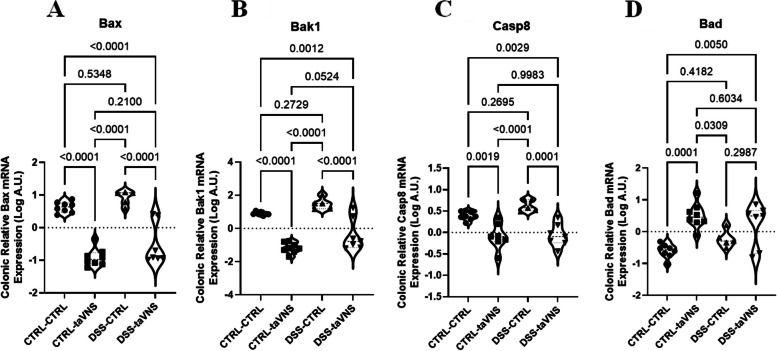
Non-invasive transcutaneous auricular vagus nerve stimulation (taVNS) decreases the colonic expression levels of pro-apoptotic markers in non-colitic and colitic mice and increases the expression level of the pro-survival marker Bad in non-colitic mice. taVNS (with stimulation parameters of 10 volts, a 500 µs pulse width, 30 s on/off duration, 10 min stimulation duration, and a frequency of 20 Hz) and no stimulation (anesthesia without any stimulation) were performed for the taVNS and CTRL (no stimulation) groups, respectively, beginning one day before the induction of acute colitis and continued daily until the mice were sacrificed. After 24 h, acute colitis was induced in the colitis (dextran sulfate sodium (DSS)) groups by adding 5% DSS to their drinking water for five days, while control groups continued to receive regular water. Colonic mRNA levels of the pro-apoptotic markers Bcl-2-associated protein x (Bax) (**A**), BCL2 antagonist/killer 1 (Bak1) (**B**), and caspase 8 (casp8) (**C**) and the pro-survival marker BCL2 associated agonist of cell death (Bad) (**D**). One-way ANOVA and multiple parametric comparison tests (Bonferroni and Šídák) were used to calculate *P* values. The significance level was set at *p* < 0.05. *n* = 5–8 mice/group. Each value is presented as the mean ± SD. This experiment was repeated at least four times

To examine whether taVNS could regulate uncontrolled apoptosis in colitis (Wan et al. 2022), the mRNA expression levels of three pro-apoptotic molecules Bax, Bak1, and Casp8, and the pro-survival molecule Bad were measured. taVNS decreased the expression of the Bax (Fig.5A, 3.31% and 8.9%, respectively), Bak1 (Fig. 5B, 1.05% and 8.26%, respectively), and Casp8 (Figs. 5C and 39.65% and 22.96%, respectively) in both non-colitic and colitic conditions, while it significantly increased the expression of the pro-survival molecule Bad in non-colitic conditions (Figs. 5D and 1587.43%).

In summary, taVNS decreased the colonic expression levels of pro-apoptotic markers and increased anti-apoptotic markers.

### DAI, macroscopic scores, and histological scores in DNBS-induced acute colitis

**Fig. 6:**
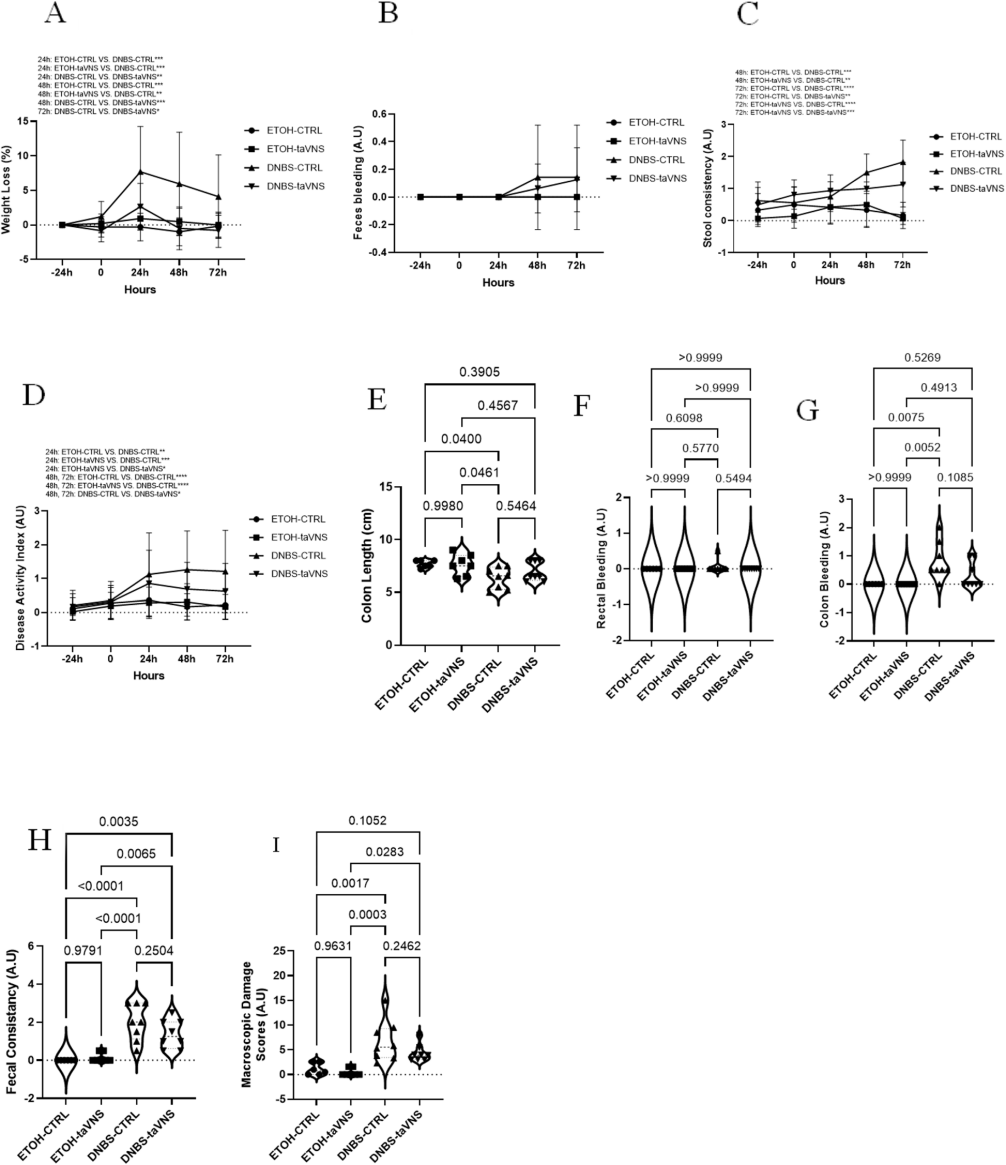
Non-invasive transcutaneous auricular vagus nerve stimulation (taVNS) improves the (dinitrobenzene sulfonic acid (DNBS)-induced acute colitis-related disease activity index (DAI) and percentage of weight loss but does not modify the macroscopic scores. taVNS (with stimulation parameters of 10 volts, a 500 µs pulse width, 30 s on/off duration, 10 min stimulation duration, and a frequency of 20 Hz) and no stimulation (anesthesia without any stimulation) were performed for the taVNS and CTRL (no stimulation) groups, respectively, beginning one day before the induction of acute colitis and continued daily until the mice were sacrificed. After 24 h, acute colitis was induced in the colitis (dinitrobenzene sulfonic acid (DNBS)) groups by a single intrarectal dose (100 µL) of 4 mg/mouse DNBS dissolved in 30% ethanol (ETOH) at time point 0, with the ETOH groups receiving 30% ethanol. Percentage of weight loss (**A**), feces bleeding (**B**), stool consistency loss (**C**), disease activity index (**D**), colon length (**E**), rectal bleeding (**F**), colon bleeding (**G**), fecal consistency (**H**), and macroscopic damage scores (**I**). One-way and Two-way ANOVA and multiple parametric comparison tests (Bonferroni and Šídák) were used to calculate *P* values. The significance level was set at *p* < 0.05. *n* = 8 mice/group. Each value is presented as the mean ± SD. This experiment was repeated at least four times

To assess if the observed alterations were specific to the DSS model, we conducted experiments utilizing the DNBS-induced colitis model. As shown in Fig. 6A, DNBS-taVNS mice had a significantly lower weight loss percentage than DNBS-CTRL mice, starting from 24 h until the end of the experiment. Additionally, non-colitic mice experienced less weight loss than DNBS-CTRL mice at the 24- and 48-hour time points (Fig. 6A). When the presence of blood in the feces was compared, no significant difference was found between the mice (Fig. 6B). Colitic mice showed significantly higher stool consistency loss compared to the non-colitic groups, starting at 48 h and continuing until the end of the experiment (Fig. 6C). At 24 h, DNBS-taVNS mice showed significantly higher DAI than ETOH-taVNS mice, and DNBS-CTRL mice had a considerably higher disease activity index than non-colitic mice (Fig. 6D). Starting from 48 h by the end of the experiment, the DAI in DNBS-taVNS and non-colitic mice was significantly lower than that in the DNBS-CTRL group (Fig. 6D). However, when all the mice upon sacrifice were compared in terms of colon length (Fig. 6E), rectal bleeding (Fig. 6F), colon bleeding (Fig. 6G), fecal consistency (Fig. 6H), and macroscopic damage scores (Fig. 6I), no impact of taVNS was observed in colitic and non-colitic mice.

**Fig. 7:**
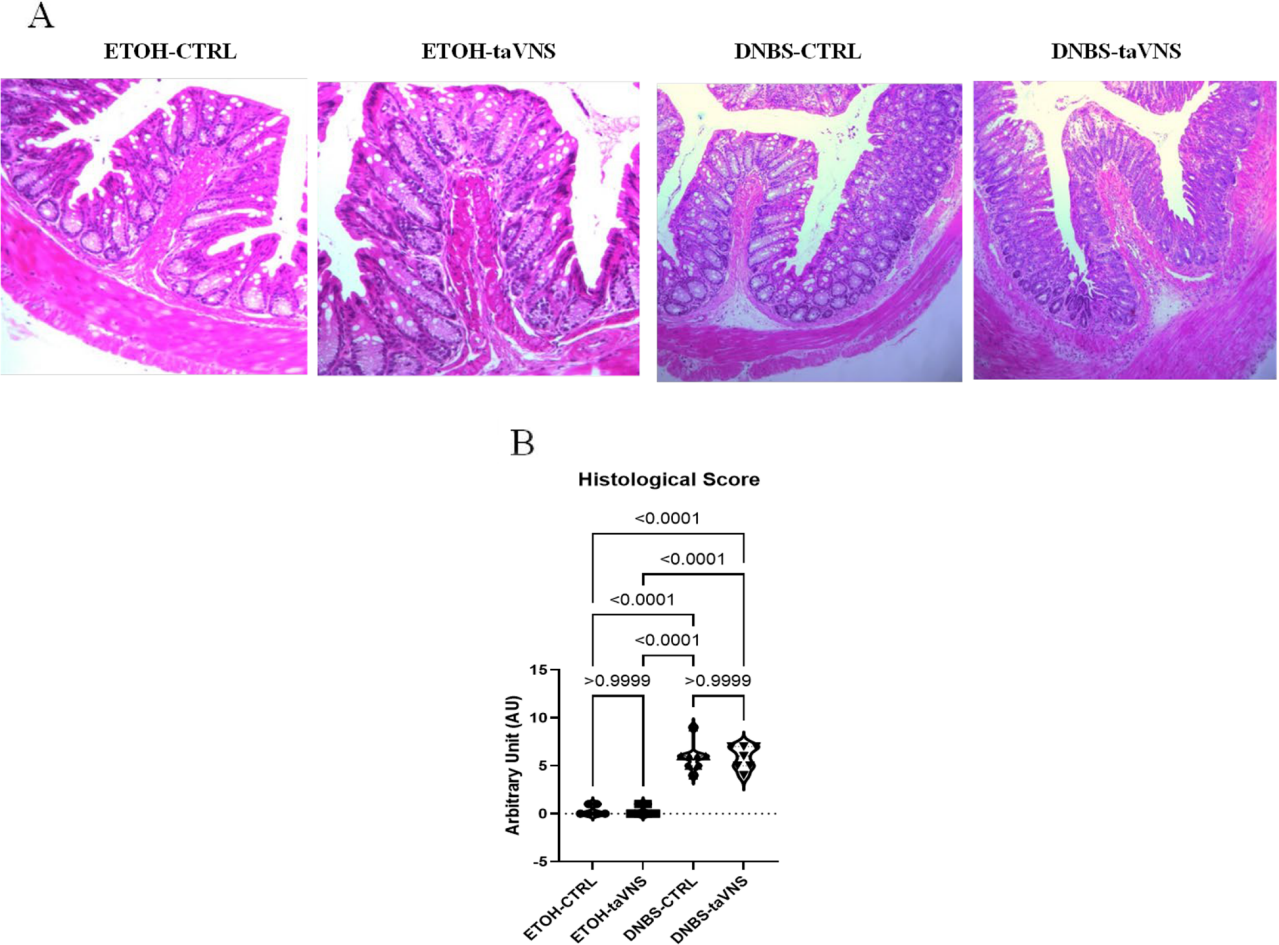
Non-invasive transcutaneous auricular vagus nerve stimulation (taVNS) does not modify the (dinitrobenzene sulfonic acid (DNBS)-induced acute colitis-related tissue and mucosal damage and histological scores. taVNS (with stimulation parameters of 10 volts, a 500 µs pulse width, 30 s on/off duration, 10 min stimulation duration, and a frequency of 20 Hz) and no stimulation (anesthesia without any stimulation) were performed for the taVNS and CTRL (no stimulation) groups, respectively, beginning one day before the induction of acute colitis and continued daily until the mice were sacrificed. After 24 h, acute colitis was induced in the colitis (dinitrobenzene sulfonic acid (DNBS)) groups by a single intrarectal dose (100 µL) of 4 mg/mouse DNBS dissolved in 30% ethanol (ETOH) at time point 0, with the ETOH groups receiving 30% ethanol. Histological appearance of hematoxylin and eosin-stained colonic tissue (40 × magnification) (**A**) and histological score (**B**). One-way ANOVA and multiple parametric comparison tests (Bonferroni and Šídák) were used to calculate *P* values. The significance level was set at *p* < 0.05. *n* = 8 mice/group. Each value is presented as the mean ± SD. This experiment was repeated at least four times

Although histological modifications such as crypt abscess, goblet cell depletion, muscle thickening, and inflammation were observed in colitic mice (Fig. 7A), no significant difference was found in taVNS colitic mice, consistent with the calculated histological score (Fig. 7B).

In summary, taVNS improved DAI in DNBS-induced acute colitis but did not significantly affect macroscopic and histological scores.

### Local anti-inflammatory effects of taVNS in DNBS-induced acute colitis

**Fig. 8:**
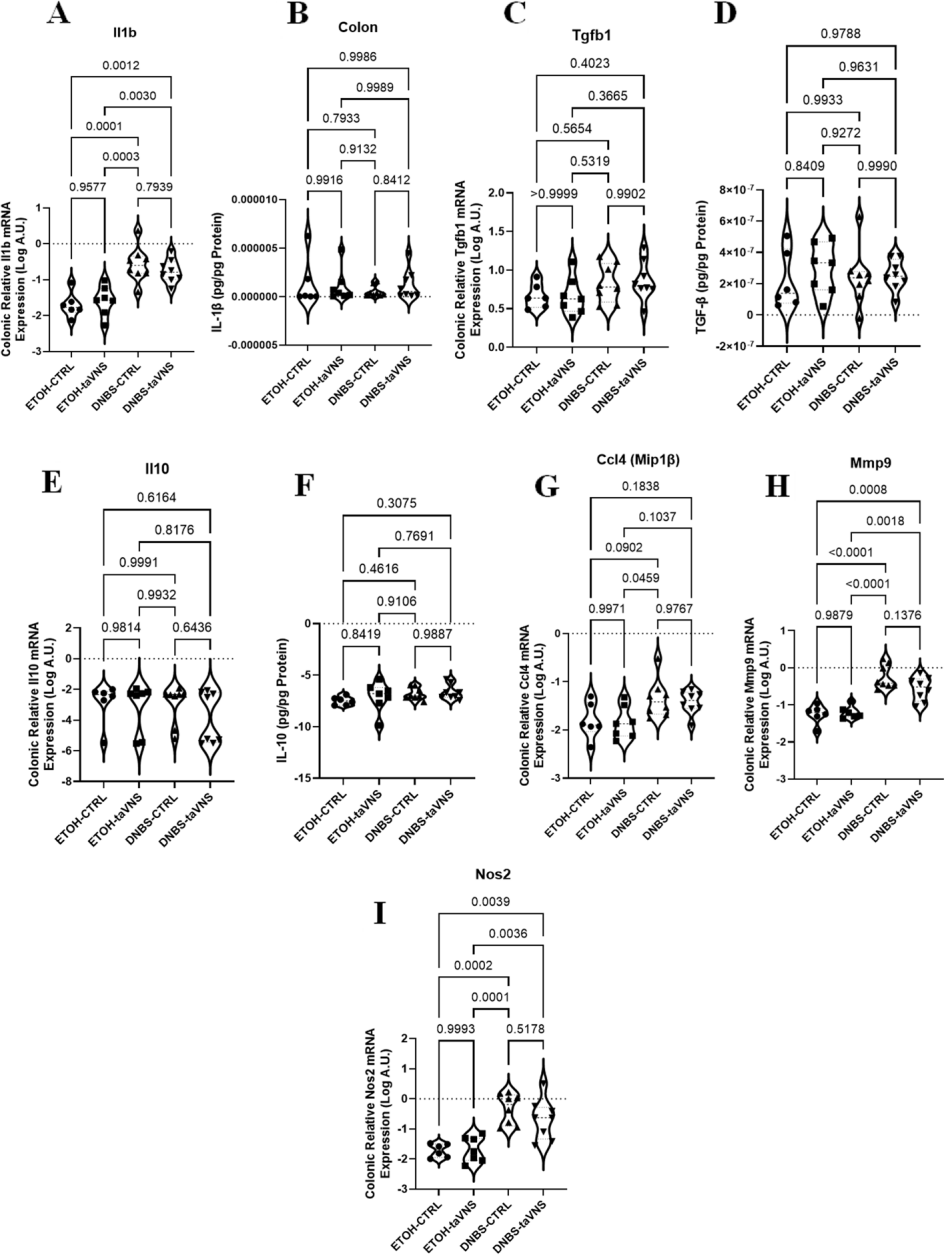
Local effects: Non-invasive transcutaneous auricular vagus nerve stimulation (taVNS) does not modify colonic expression levels of pro-inflammatory and anti-inflammatory markers in DNBS colitic and non-colitic mice. taVNS (with stimulation parameters of 10 volts, a 500 µs pulse width, 30 s on/off duration, 10 min stimulation duration, and a frequency of 20 Hz) and no stimulation (anesthesia without any stimulation) were performed for the taVNS and CTRL (no stimulation) groups, respectively, beginning one day before the induction of acute colitis and continued daily until the mice were sacrificed. After 24 h, acute colitis was induced in the colitis (dinitrobenzene sulfonic acid (DNBS)) groups by a single intrarectal dose (100 µL) of 4 mg/mouse DNBS dissolved in 30% ethanol (ETOH) at time point 0, with the ETOH groups receiving 30% ethanol. Colonic mRNA (**A**) and protein (**B**) levels of interleukin (IL)−1β, mRNA (**C**) and protein (**D**) levels of transforming growth factor (TGF)-β, mRNA (**E**) and protein (**F**) levels of IL-10, mRNA levels of macrophage inflammatory protein 1β (Mip1β) or chemokine (C-C motif) ligands 4 (CCL4) (**G**), matrix metalloproteinases 9 (MMP9) (**H**), and nitric oxide synthase 2 (Nos2) (**I**). One-way ANOVA and multiple parametric comparison tests (Bonferroni and Šídák) were used to calculate *P* values. The significance level was set at *p* < 0.05. *n* = 2–8 mice/group. Each value is presented as the mean ± SD. This experiment was repeated at least four times

Colonic expression levels of some selected inflammatory and anti-inflammatory cytokines, chemokines, MMPs, and neutrophil/macrophage-associated inflammatory markers were examined. No significant differences were observed when colonic protein and mRNA levels of IL-1β, TGF-β1, and IL-10 were compared between colitic and non-colitic conditions (Fig. 8A and F). Additionally, no changes were seen in the mRNA levels of Mip1β (CCL4), MMP9, and Nos2 (Fig. 8G and I) in any of the groups of mice.

In summary, no modifications were found in colonic levels of inflammatory and anti-inflammatory markers involved in the development or prevention of DNBS-induced acute colitis.

### Systemic anti-inflammatory effects of taVNS in DNBS-induced acute colitis

**Fig. 9:**
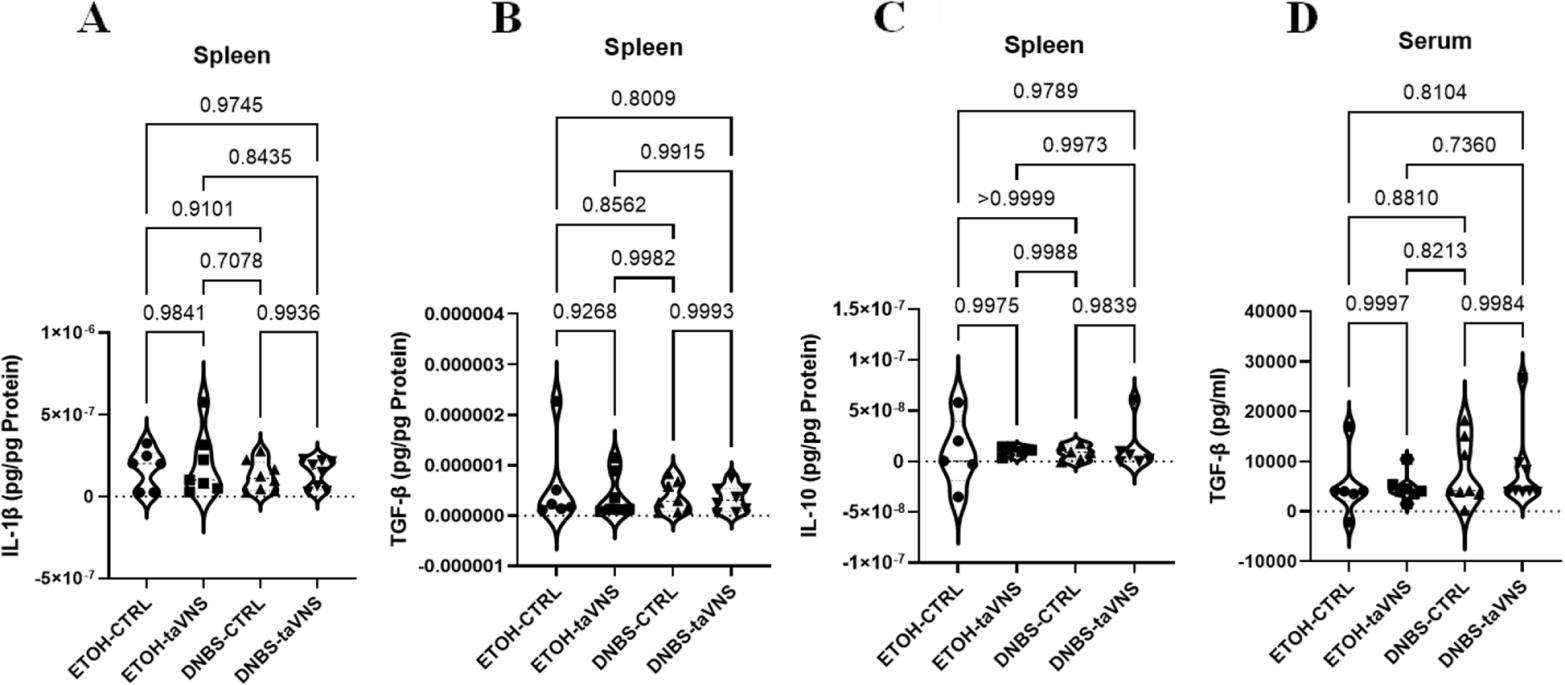
Systemic effects: Non-invasive transcutaneous auricular vagus nerve stimulation (taVNS) does not modify splenic and serum levels of pro-inflammatory and anti-inflammatory markers in DNBS colitic and non-colitic mice. taVNS (with stimulation parameters of 10 volts, a 500 µs pulse width, 30 s on/off duration, 10 min stimulation duration, and a frequency of 20 Hz) and no stimulation (anesthesia without any stimulation) were performed for the taVNS and CTRL (no stimulation) groups, respectively, beginning one day before the induction of acute colitis and continued daily until the mice were sacrificed. After 24 h, acute colitis was induced in the colitis (dinitrobenzene sulfonic acid (DNBS)) groups by a single intrarectal dose (100 µL) of 4 mg/mouse DNBS dissolved in 30% ethanol (ETOH) at time point 0, with the ETOH groups receiving 30% ethanol. Splenic protein levels of interleukin (IL)−1β (**A**), TGF-β (**B**), and IL-10 (**C**) and serum levels of TGF-β (**D**). One-way ANOVA and multiple parametric comparison tests (Bonferroni and Šídák) were used to calculate *P* values. The significance level was set at *p* < 0.05. *n* = 5–8 mice/group. Each value is presented as the mean ± SD. This experiment was repeated at least four times

Splenic protein and serum levels of pro-inflammatory and anti-inflammatory cytokines, including IL-1β, TGF-β, and IL-10, were measured, but no changes were observed in these cytokines in colitic or non-colitic conditions (Fig. 9A and C). Additionally, the absence of changes in serum levels of TGF-β (Fig. 9D) was observed in both colitic and non-colitic conditions.

In summary, taVNS appeared to have no significant impact on splenic or serum levels of inflammatory and anti-inflammatory markers associated with developing or preventing DNBS-induced acute colitis.

### The colonic expression levels of apoptotic markers in DNBS-induced acute colitis

**Fig. 10:**
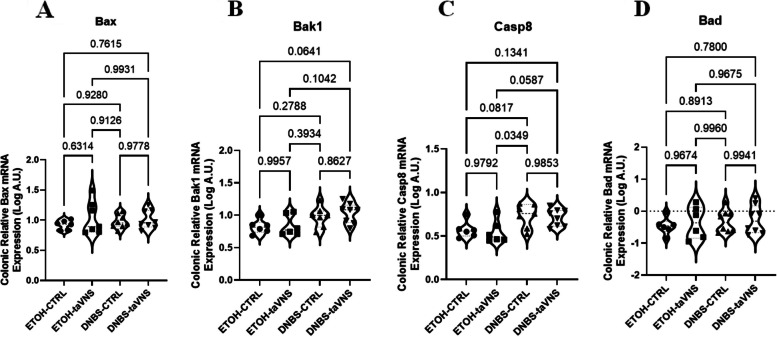
Non-invasive transcutaneous auricular vagus nerve stimulation (taVNS) does not modify colonic expression levels of pro-apoptotic and pro-survival markers in the (dinitrobenzene sulfonic acid (DNBS)-induced colitic and non-colitic mice. taVNS (with stimulation parameters of 10 volts, a 500 µs pulse width, 30 s on/off duration, 10 min stimulation duration, and a frequency of 20 Hz) and no stimulation (anesthesia without any stimulation) were performed for the taVNS and CTRL (no stimulation) groups, respectively, beginning one day before the induction of acute colitis and continued daily until the mice were sacrificed. After 24 h, acute colitis was induced in the colitis (dinitrobenzene sulfonic acid (DNBS)) groups by a single intrarectal dose (100 µL) of 4 mg/mouse DNBS dissolved in 30% ethanol (ETOH) at time point 0, with the ETOH groups receiving 30% ethanol. Colonic mRNA levels of the pro-apoptotic markers Bcl-2-associated protein x (Bax) (**A**), BCL2 antagonist/killer 1 (Bak1) (**B**), and caspase 8 (casp8) (**C**) and the pro-survival marker BCL2 associated agonist of cell death (Bad) (**D**). One-way ANOVA and multiple parametric comparison tests (Bonferroni and Šídák) were used to calculate *P* values. The significance level was set at *p* < 0.05. *n* = 8 mice/group. Each value is presented as the mean ± SD. This experiment was repeated at least four times

The mRNA expression levels of apoptosis-related markers, including pro-apoptotic molecules Bax (Fig. 10A), Bak1 (Fig. 10B), and Casp8 (Fig. 10C), as well as the pro-survival molecule Bad (Fig. 10D), were assessed in DNBS colitic and non-colitic conditions with no significant changes observed.

In conclusion, taVNS did not affect apoptotic markers in DNBS-induced acute colitis.

## Discussion

Although a recent clinical trial has reported beneficial effects of taVNS in reducing symptoms in pediatric IBD patients (Sahn et al. 2023), to our knowledge, this study is the first to evaluate both systemic and local effects of non-invasive stimulation of the auricular cymba concha on the progression of experimental acute colitis induced by DSS and DNBS, as well as on the expression of apoptotic and pro- and anti-inflammatory markers. Our results demonstrated that taVNS significantly improved the DAI, macroscopic scores, and histological scores in the UC-like DSS-induced colitis model and partially mitigated weight loss and DAI in CD-like DNBS-induced colitis model. These findings align with previously published studies using invasive VNS in different animal models of IBD. For example, studies on 2,4,6-trinitrobenzene sulfonic acid (TNBS)-induced CD-like colitis in rats, as well as DSS-induced UC-like colitis and oxazolone-induced colitis in mice, have reported that VNS reduced body weight loss and improved disease activity in the animals (Meroni et al. 2018, Sun et al. 2013, Payne et al. 2019). Our findings indicate that the influence of taVNS is contingent on the specificity of the model used. This observation aligns with previous research, which has demonstrated that ACh exerts dual effects depending on the subtype of IBD, whether T helper type 2 (Th2)-driven UC or Th1-driven CD (Serafini et al. 2022). Although the exact mechanisms remain unclear, Galitovskiy and colleagues (Galitovskiy et al. 2011) developed mouse models of Th1 and Th2 inflammation, showing that in the Th2 model, nicotine, which mimics ACh, induced α7nAChR expression on CD4^+^T cells, leading to an increase in regulatory T cells and a decrease in inflammation (Galitovskiy et al. 2011). In contrast, the Th1 inflammation model exhibited no increase in receptor expression or reduction in inflammation (Galitovskiy et al. 2011). Considering that CD is immunologically classified as a Th1-dominant condition and UC as Th2-dominant, it is likely that the immunomodulatory effects of vagus nerve activity are mediated via distinct pathways in each disease (Brand 2009, Heller et al. 2005). While subclinical autonomic dysregulation is more frequently observed in UC patients than in those with CD, VNS has also shown early clinical promise in treating CD (Mogilevski et al. 2019). These findings imply that enhancing vagal tone in CD could promote a Th2-biased immune response, whereas in patients with UC, reestablishing the equilibrium between sympathetic and vagal activity might be more crucial (Mogilevski et al. 2019). Moreover, the model-specific effects of taVNS in colitis may be linked to variations in disease severity and inflammatory patterns between the UC-like DSS model, characterized by superficial mucosal inflammation confined to the colon, and the CD-like DNBS model, which involves transmural inflammation throughout the gastrointestinal tract (Roushan et al. 2019).

The pathogenesis of IBD involves complex interactions between pro-inflammatory and anti-inflammatory cytokines and different immune cells (Singh et al. 2016). Neutrophils are recruited to the inflamed gut following epithelial damage in response to chemokines produced by cluster of differentiation 4 (CD4) T cells, epithelial cells, or myofibroblasts, contributing to tissue damage by releasing MMPs, inducible nitric oxide synthase (iNOS)-derived nitric oxide, and pro-inflammatory cytokines (Kang et al. 2022). TNF-α and IL-1β promote monocyte-to-macrophage differentiation and pathological T-cell responses, with TNF-α further driving Th17 differentiation and epithelial damage in chronic inflammation (Weigmann and Neurath 2016, Kaur and Goggolidou 2020, Ho et al. 2020). Anti-inflammatory cytokines like IL-10 regulate immune responses by enhancing regulatory T cell activity, and loss of IL-10 signaling exacerbates IL-1β production, Th17 differentiation, and colitis (Li et al. 2015, Wei et al. 2020). The DSS-induced colitis model shows that IL-10-deficient macrophages exhibit increased NO production, leading to inflammation (Wei et al. 2020, Li et al. 2014). IL-10 also regulates nuclear factor kappa-light-chain-enhancer of activated B cells (NF-κB) expression, with aberrant NF-κB activation and reduced IL-10 levels linked to IBD (Kaur and Goggolidou 2020). TGF-β also significantly maintains intestinal homeostasis by inducing Tregs and supporting epithelial integrity (Babyatsky et al. 1996). VNS has a potential anti-inflammatory effect on IBD by activating intestinal CAP, where ACh released from the ENS inhibits pro-inflammatory cytokine production and increases IL-10 production by interacting with immune cells (Bonaz et al. 2021, Zheng et al. 2024). VNS also activates a complex splenic CAP by releasing ACh in the celiac ganglia, which interacts with the splenic nerve to induce norepinephrine (NE) release in the spleen (Rosas-Ballina et al. 2008). NE then activates splenic CD4^+^T cells, which produce ACh through a non-neuronal cholinergic pathway (Rosas-Ballina et al. 2008). This ACh reduces the production of inflammatory cytokines, especially TNF-α, by interacting with the α7nAChR found on macrophages in the spleen (Olofsson et al. 2012). These activated T cells can also migrate to non-innervated areas, reducing local and systemic inflammation (Bonaz et al. 2021). Animal studies using invasive VNS have shown decreased levels of inflammatory markers related to the pathogenesis of colitis, such as TNF-α, IL-1β, and iNOS activity (Meroni et al. 2018, Sun et al. 2013, Payne et al. 2019). In line with this, our findings indicated that taVNS significantly suppressed both local and systemic DSS-induced colitis-associated inflammation by decreasing the levels of inflammatory cytokines, chemokines, and neutrophil/macrophage-related markers while increasing protective anti-inflammatory cytokines such as IL-10 and TGF-β. However, it is essential to note that the same effects were not observed in the DNBS colitis model. This suggests that the effects of VNS on inflammation might be model-dependent and influenced by the cellular background of IBD, as previously discussed (Mogilevski et al. 2019). In addition, as mentioned above, the model-specific effects of taVNS in colitis may be linked to variations in disease severity and inflammatory patterns between the UC-like DSS and CD-like DNBS models. In line with that, a study using invasive VNS in a CD-like rat model showed that the effectiveness of VNS in decreasing inflammatory markers was higher in regions above the lesion than at the lesion itself. This suggests that the severity and extent of tissue damage should be considered when evaluating the effectiveness of VNS (Meregnani et al. 2011).

Most alterations observed in the markers mentioned, including IL-1β and TNF-α, primarily occur at the mRNA level rather than the protein level. This underscores the complexity of the mRNA-protein relationship, as it is regulated by various factors beyond transcript abundance (Liu et al. 2016). These include the efficiency of translation, post-transcriptional and post-translational modifications, protein stability, temporal delays in synthesis, and spatial distribution (Liu et al. 2016). Sampling location is also crucial, as exporting proteins, particularly cytokines, can create spatial separation from their corresponding mRNA, complicating direct comparisons from the same sample site (Liu et al. 2016). Additionally, sampling timing is pivotal due to the delay between mRNA transcription and protein synthesis, coupled with differing half-lives of mRNA and proteins (Liu et al. 2016).

Surprisingly, our data demonstrate that in the absence of colitis, taVNS can effectively reduce splenic TNF-α levels while increasing colonic and splenic IL-10 and TGF-β, suggesting that taVNS may help maintain homeostasis by lowering sympathetic nervous system activity while increasing vagus nerve activity. This is supported by human studies (Clancy et al. 2014, Tsaava et al. 2020)demonstrating that auricular nerve stimulation can reduce sympathetic activity, which is commonly linked to increased systemic levels of inflammatory cytokines such as TNF-α, contrasting with the parasympathetic nervous system, with the vagus nerve as a central element, plays a different role (Pongratz and Straub 2023).

Apoptosis, particularly its intrinsic pathway, is predominantly regulated by the Bcl-2 protein family, which includes pro-apoptotic proteins (such as Bax and Bak1) and anti-apoptotic or pro-survival factors like Bad (Wan et al. 2022). Interestingly, although Bad is generally recognized for its pro-apoptotic function, it has been observed to inhibit cell death in specific cell types under particular circumstances (So et al. 2004). Moreover, caspase 8 is pivotal in the inflammatory process, given its involvement at multiple stages of apoptosis (Wan et al. 2022). In studies involving colitis mouse models and patients with UC, there is a notable increase in the expression of pro-apoptotic molecules Bax and Bak, along with caspase family members like casp8 and casp3, compared to control groups (Nunes et al. 2014). This elevated expression has also been linked to inflammatory cytokine-induced epithelial apoptosis in CD, contributing to the epithelial barrier’s deterioration (Nunes et al. 2014). Previous studies using VNS in mouse models of colitis have reported that invasive VNS promoted tissue repair by enhancing epithelial barrier integrity and reducing apoptosis (Meroni et al. 2021). Our results also suggest that taVNS exerts a protective effect in both DSS-colitis mice and non-colitic conditions by reducing the levels of pro-apoptotic molecules.

Additionally, it enhances the expression of the pro-survival molecule Bad, although this increase is observed only under non-colitic conditions. These results are consistent with earlier reports demonstrating the anti-apoptotic effects of VNS, which have been linked to the downregulation of NF-κB and myosin light chain kinase via the α7nAChR pathway, thereby strengthening the intestinal epithelial barrier (Zhou et al. 2013). However, taVNS did not significantly alter the expression of these apoptosis-related molecules in DNBS-induced colitic and non-colitic mice, likely due to the model-dependent effects of taVNS. The similarity between DNBS-colitis mice and non-colitic conditions regarding the apoptotic markers may be attributed to the inclusion of 30% ethanol in the intrarectal injections administered to both groups. Indeed, ethanol administration, known to compromise the integrity of the mucosal barrier in general (Elamin et al. 2014), is essential for disrupting this barrier to allow DNBS to penetrate the lamina propria, where it binds to local colonic proteins and triggers subsequent immune responses (Morampudi et al. 2014).

### Limitations

Although taVNS exhibits anti-inflammatory and protective properties, it is important to acknowledge certain limitations associated with our findings. Recent findings suggest that the auricular VNS (AVNS) zone, including the cymba concha, used in non-invasive stimulation, may be innervated by non-vagal nerves, potentially activating non-vagal nuclei rather than the NTS (Yap et al. 2020). This possibility necessitates a more comprehensive analysis of the central and peripheral neural circuits involved in taVNS (Hesampour et al. 2024). To determine the pathways implicated from the central nervous system to the colon and to confirm the importance of the vagus nerve and spleen in mediating the anti-inflammatory and preventive effects observed in this study, future research should include bilateral vagotomy, splenectomy, and splenic neurectomy to assess whether the beneficial effects of taVNS can be abolished by disrupting these pathways.

Since the stimulation parameters used in our study could also activate the HPA axis and contribute to the anti-inflammatory effects of taVNS observed in the results, we measured serum glucocorticoid levels to assess the involvement of the HPA axis and could not see any modifications. However, further investigations are needed to confirm this finding.

The duration and frequency of VNS in mice can significantly influence experimental results. In the literature, VNS treatments have been employed with varying durations, such as 10-minute daily sessions with 30-second on/off intervals over 5 days, in IBD models in both mice and rats (Meregnani et al. 2011, Bonaz et al. 2017). However, identifying the optimal VNS duration and frequency for acute colitis in mice requires careful consideration of factors such as colitis severity, the specific mouse model, and the intended outcomes. In our study, the pre-established UC-like DSS colitis model involved a 5-day DSS treatment. To assess the efficacy of taVNS under the stimulation parameters outlined in the methods section, we initiated taVNS or the control (no stimulation) one day before DSS colitis induction. A similar approach was used for the DNBS group. In this 4-day experiment, taVNS or no stimulation began one day before DNBS colitis induction, followed by three additional days post-DNBS intrarectal injection. Nevertheless, it is important to acknowledge that differences in stimulation duration (6 days for the DSS model vs. 4 days for the DNBS model) and the severity of the colitis models represent limitations in fully assessing the effectiveness of taVNS in IBD.

The setting used could have also impacted the results. During the 10-minute daily stimulation for each mouse, anesthesia was maintained with 2.5% isoflurane. Isoflurane is commonly employed in anesthesia procedures due to its dose controllability, minimal animal stress induction, and ease of use (Silverman et al. 2018). However, it is important to note that isoflurane may exert immunomodulatory effects in experimental models of VNS (Silverman et al. 2018, Picqet al. 2013). Specifically, isoflurane-induced anesthesia can lead to dose-dependent inhibitory effects on nerve activity and conduction velocity (Silverman et al. 2018).

Another limitation of this study, which should be acknowledged, is that the small size of the collected colon tissues resulted in insufficient homogenate for most samples. Consequently, we were restricted to measuring a subset of selected inflammatory and anti-inflammatory markers and could not assess the neutrophil-associated marker myeloperoxidase (MPO). Although previous studies have demonstrated that VNS significantly reduces MPO levels in colon tissue, given MPO’s critical role in assessing colitis disease activity (Meregnani et al. 2011), it would be valuable to measure MPO expression in our DSS and DNBS colitis models to determine whether these findings are consistent with earlier reports.

Additionally, this study focused exclusively on male mice to establish the initial parameters and evaluate the preventive effects of taVNS in the context of acute colitis. While this approach allows for controlling the physiological variability associated with the estrous cycle in female mice, it limits the generalizability of the findings regarding sex-related differences in IBD and taVNS effects. The role of sex-related differences in IBD is complex and not well understood, with clinical studies reporting conflicting results regarding IBD incidence, treatment response, and progression between sexes (Rustgi et al. 2020). Furthermore, studies have indicated functional differences between males and females in vagus nerve activity and parasympathetic responses, which could impact the outcomes of taVNS (Matsumoto et al. 2007). Therefore, future studies are warranted to include female mice to fully explore the sex-specific effects of taVNS in colitis models.

## Conclusions

The findings of this study demonstrate that taVNS significantly mitigates DSS-induced colitis development in mice, as evidenced by the decreased expression of pro-inflammatory and pro-apoptotic markers and increased levels of anti-inflammatory markers. This suggests that taVNS is crucial in controlling gut inflammation in colitic mice. These immunomodulatory effects seem to be a systemic process, likely mediated through the splenic CAP, which aids in preventing and controlling colitis-associated systemic inflammation. Simultaneously, these effects are probably locally regulated by efferent vagal fibers that directly stimulate enteric neurons to produce specific neurotransmitters, thereby reducing local inflammation and tissue damage. The observed benefits of taVNS, including the upregulation of anti-inflammatory cytokines and downregulation of pro-inflammatory cytokines, also underscore the importance of VNS in non-inflammatory conditions and homeostasis. However, further studies are necessary to confirm these findings and to elucidate the cellular and molecular mechanisms underlying the contributions of the splenic and intestinal CAP and α7nAChR in both acute colitis and steady-state conditions. It is important to note that the local and systemic benefits of taVNS appear to be model-dependent. These results suggest further research to elucidate the differences in cellular and molecular mediators of taVNS between the DSS and DNBS colitis models. Furthermore, it is recommended to assess the impact of taVNS on the spatial distribution of various immune cell subsets within the spleen and across different sections of the colon tissue. This approach will help to further elucidate the cellular mediators involved in taVNS efficacy in mouse models of UC-like colitis.

## Data Availability

The datasets used and/or analyzed during the current study are available from the corresponding author upon reasonable request.

## Abbreviations

Ach: Acetylcholine
AVNS: Auricular vagus nerve stimulation
Bad: BCL2 associated agonist of cell death
Bak1: BCL2 antagonist/killer 1
Bax: Bcl-2-associated protein x
CAP: Cholinergic anti-inflammatory pathway
Casp8: Caspase 8
CCL: Chemokine (C-C motif) ligand
CD: Cluster of differentiation
CD: Crohn’s disease
ChAT: Choline acetyltransferase
CTRL: Control
DAI: Disease activity index
DMV: Dorsal motor nucleus of the vagus nerve
DNBS: Dinitrobenzene sulfonic acid
DSS: Dextran sulfate sodium
ELISA: Enzyme-linked immunosorbent assay
ENS: Enteric nervous system
ETOH: Ethanol
HPA: Hypothalamic-pituitary-adrenal axis
IBD: Inflammatory bowel disease
IL: Interleukin
iNOS: Inducible nitric oxide synthase
Mip: Macrophage inflammatory protein
MMP: Matrix metalloproteinases
MMØs: Muscularis macrophages
MPO: Myeloperoxidase
NE: Norepinephrine
NF-κB: Nuclear factor kappa-light-chain-enhancer of activated B cells
Nos: Nitric oxide synthase
NTS: Nucleus tractus solitarii
PBS: Phosphate-buffered saline
qRT-PCR: Quantitative real-time reverse-transcription polymerase chain reaction
taVNS: Transcutaneous vagus nerve stimulation
TBP: TATA-box binding protein
TGF: Transforming growth factor
Th: T helper
TNBS: 2,4,6-trinitrobenzene sulfonic acid
TNF: Tumor necrosis factor
UC: Ulcerative colitis
α7nAChR: α7 nicotinic ACh receptor

## Declarations

JEG has served as a member of the scientific medical advisory committee for Crohn’s and Colitis Canada and as a member and chair of the research advisory committee for Research Manitoba.
